# Determination of intracellular protein–ligand binding affinity by competition binding in-cell NMR

**DOI:** 10.1107/S2059798321009037

**Published:** 2021-09-27

**Authors:** Enrico Luchinat, Letizia Barbieri, Matteo Cremonini, Matteo Pennestri, Alessio Nocentini, Claudiu T. Supuran, Lucia Banci

**Affiliations:** a CERM – Magnetic Resonance Center, Università degli Studi di Firenze, Via Luigi Sacconi 6, 50019 Sesto Fiorentino, Italy; b Dipartimento Neurofarba, Università degli Studi di Firenze, Via Ugo Schiff 6, 50019 Sesto Fiorentino, Italy; c CIRMMP – Consorzio Interuniversitario Risonanze Magnetiche di Metalloproteine, Via Luigi Sacconi 6, 50019 Sesto Fiorentino, Italy; d Dipartimento di Chimica, Università degli Studi di Firenze, Via della Lastruccia 3, 50019 Sesto Fiorentino, Italy; e Pharmaceutical Business Unit, Bruker UK Limited, Banner Lane, Coventry CV4 9GH, United Kingdom

**Keywords:** competition binding, in-cell NMR, real-time NMR, bioreactor, carbonic anhydrase inhibitors

## Abstract

Structure-based drug development suffers from high attrition rates due to the poor activity of lead compounds in cellular and animal models caused by low cell penetrance, off-target binding or changes in the conformation of the target protein in the cellular environment. The latter two effects cause a change in the apparent binding affinity of the compound, which is indirectly assessed by cellular activity assays. To date, direct measurement of the intracellular binding affinity remains a challenging task. In this work, in-cell NMR spectroscopy was applied to measure intracellular dissociation constants in the nanomolar range by means of protein-observed competition binding experiments. Competition binding curves relative to a reference compound could be retrieved either from a series of independent cell samples or from a single real-time NMR bioreactor run. The method was validated using a set of sulfonamide-based inhibitors of human carbonic anhydrase II with known activity in the subnanomolar to submicromolar range. The intracellular affinities were similar to those obtained *in vitro*, indicating that these compounds selectively bind to the intracellular target. In principle, the approach can be applied to any soluble intracellular target that gives rise to measurable chemical shift changes upon ligand binding.

## Abstract 2

Intracellular protein–ligand dissociation constants are measured by in-cell NMR spectroscopy by means of competition binding experiments with respect to a reference ligand. The method is applied to a set of carbonic anhydrase inhibitors, revealing intracellular binding with nanomolar affinity.

## 1. Introduction   

Structure-based drug-design approaches rely on knowledge of the three-dimensional structure of the target protein to develop effective drugs. The target structure is fundamental in the initial steps of drug development, from initial screening and hit identification to the optimization of lead compounds. In the preclinical studies that follow, the best-performing candidates *in vitro* are screened for in-cell or *in vivo* activity by cell-based assays *in vitro* and/or in animal models. At this stage of drug development, the efficacy of the compounds is evaluated from enzymatic assays on cell cultures, or more indirectly from other cellular responses, such as cell death, proliferation, invasiveness or metabolic activity (Hughes *et al.*, 2011 ▸; Kepp *et al.*, 2011 ▸). In these trials, there is often no direct readout of the protein–ligand interaction, and of the binding affinity, in the cellular environment. This loss of information at the molecular level increases the risk of promoting to the later phases of drug development compounds that, despite being highly active *in vitro*, fail in the preclinical phases, or even later in the clinical trials, due to lack of intracellular activity or due to off-target activity causing unwanted side effects. A method to quantitatively measure the affinity of a ligand towards its intracellular target could therefore provide precious information on the efficacy of candidate drugs within the physiological environment of a living cell, and provide mechanistic insight on the cellular response, or lack thereof, to drug treatment at an earlier phase of drug development, thereby increasing the success rate in the later phases.

Nuclear magnetic resonance (NMR) spectroscopy can provide high-resolution chemical and structural information on protein–ligand interactions in complex solutions in a non­destructive way. As such, it is the only high-resolution structural technique that can be applied to living cells at physiological temperatures (Luchinat & Banci, 2018*b* ▸; Siegal & Selenko, 2019 ▸). The approach, termed in-cell NMR, can indeed be applied to study the structure of proteins and nucleic acids directly in living cells (Sakakibara *et al.*, 2009 ▸; Inomata *et al.*, 2009 ▸; Theillet *et al.*, 2016 ▸; Dzatko *et al.*, 2018 ▸; Tanaka *et al.*, 2019 ▸; Broft *et al.*, 2021 ▸), their interaction with the cellular environment or with specific partners (Majumder *et al.*, 2015 ▸; Smith *et al.*, 2016 ▸; Luchinat *et al.*, 2017 ▸), and the binding of small cofactors and metal ions (Luchinat & Banci, 2018*a* ▸; Capper *et al.*, 2018 ▸; Polykretis *et al.*, 2019 ▸). In recent years, in-cell NMR has been shown to be a promising approach in the context of drug development, as it provides a direct, nondestructive measure of protein–ligand and nucleic acid–ligand interactions inside bacterial and human cells (DeMott *et al.*, 2018 ▸; Krafcikova *et al.*, 2019 ▸; Luchinat, Barbieri, Cremonini *et al.*, 2020*a* ▸). We have previously applied protein-observed in-cell NMR to perform small-scale ligand screening in human cells, in which the amounts of free and bound protein were measured quantitatively as a function of dose and time of treatment (Luchinat, Barbieri, Cremonini *et al.*, 2020*a* ▸,*b* ▸). For each ligand, pharmacologically relevant parameters were obtained such as cell penetrance and ligand–protein complex stability over time. Additionally, dose-dependent binding data provided an estimate of the apparent in-cell binding affinity. However, in order to accurately determine the dissociation constants of strong binders (*i.e.* ligands showing *K*_d_ values lower than submicromolar) by protein-observed NMR, competition binding experiments must be employed (Dalvit *et al.*, 2002 ▸). Here, we demonstrate that protein-observed in-cell NMR can be applied to perform intracellular competition binding experiments. This method allows determination of the intracellular affinity of ligands with *K*_d_ values in the nanomolar range, relative to the *K*_d_ of a reference compound. We provide two alternative approaches to obtain intracellular competition binding curves: (i) by conventional ‘closed-tube’ in-cell NMR, in which several independent cell samples, each of which is treated with two competing ligands at different doses, are analyzed separately for a short acquisition time to preserve cell viability, and (ii) by time-resolved in-cell NMR through the use of an NMR bio­reactor (Kubo *et al.*, 2013 ▸; Breindel *et al.*, 2018 ▸; Luchinat, Barbieri, Campbell *et al.*, 2020 ▸), in which a single sample of cells is kept viable and metabolically active for a prolonged period of time, during which a test ligand is added at increasing concentrations in a stepwise manner together with a reference compound kept at a constant concentration.

To validate the method, we measured the intracellular affinity of a set of strong ligands towards the second isoform of human carbonic anhydrase (CA II). Carbonic anhydrases (CAs; EC 4.2.1.1) are ubiquitous enzymes that catalyze the hydration of CO_2_ with H_2_O to generate 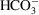 and H^+^ (Supuran, 2008 ▸, 2021 ▸). All 15 human isoforms of CA belong to the α-class and bind a catalytic zinc ion through three conserved histidine residues and a water molecule/hydroxide anion in the active site (Supuran, 2016 ▸). Many of these isoforms, which have similar structural properties but differing subcellular localizations, catalytic activities and responses to exogenous molecules, have been implicated in several pathological states, such as epilepsy, glaucoma, cardiovascular diseases and cancer (Mboge *et al.*, 2018 ▸; Nocentini & Supuran, 2019 ▸). Therefore, these proteins are important drug targets for which it is critical to reliably measure the intracellular binding affinity in order to develop novel compounds with high *in vivo* selectivity towards specific isoforms (Angeli *et al.*, 2020 ▸). The compounds analyzed here are well characterized sulfonamide-based CA inhibitors with known cellular activity that exhibit inhibitory constants (*K*_i_ values) in the subnanomolar to sub­micromolar range. Both in-cell NMR approaches provided similar intracellular *K*_d_ values, which overall were consistent with the *K*_d_ values obtained for the same ligands by competition binding *in vitro*.

## 2. Materials and methods   

### 2.1. Expression and purification of recombinant CA II   

Recombinant CA II for *in vitro* experiments was prepared following a modification of an existing protocol (Cerofolini *et al.*, 2017 ▸). Briefly, a 1 l cell culture of *Escherichia coli* BL21(DE3) Codon Plus RipL cells (Stratagene) transformed with a pCAM plasmid containing the gene encoding CA II without additional tags was grown overnight at 37°C in Luria–Bertani (LB) medium supplemented with 2 g l^−1^ glucose, harvested and resuspended in 1 l ^15^N-labeled M9 medium. ZnSO_4_ was added to the culture to a final concentration of 500 µ*M*. Protein expression was induced with 1 m*M* isopropyl β-d-1-thiogalactopyranoside, and after 5 h at 37°C the cells were harvested and resuspended in 20 m*M* Tris pH 8 for lysis. The cleared lysate was loaded onto a nickel-chelating HisTrap (GE Healthcare) 5 ml column, exploiting the fact that human CA II binds to metal-loaded resins even in the absence of a histidine tag (Banerjee *et al.*, 2004 ▸). The protein was eluted with a linear gradient of 20 m*M* Tris pH 8, 500 m*M* imidazole. The fractions containing pure CA II were collected. Finally, the protein was exchanged into NMR buffer [phosphate-buffered saline (PBS) pH 7.4 (Gibco) supplemented with 10% D_2_O].

### 2.2. Human cell cultures and transfection   

HEK 293T cells (ATCC CRL-3216) were maintained in Dulbecco’s Modified Eagle Medium (DMEM) high glucose (Gibco) supplemented with l-glutamine, antibiotics (penicillin and streptomycin) and 10% fetal bovine serum (FBS; Gibco) in uncoated 75 cm^2^ plastic flasks and incubated at 37°C and 5% CO_2_ in a humidified atmosphere. The cells were transiently transfected using branched polyethylenimine (PEI) following a previously reported protocol (Aricescu *et al.*, 2006 ▸; Barbieri *et al.*, 2016 ▸). The cells were transfected with a 1:2 DNA:PEI mixture (25 µg DNA per flask and 50 µg PEI per flask) containing a vector for high-level constitutive expression of human CA II (pHL-CAII) obtained as described previously (Luchinat, Barbieri, Cremonini *et al.*, 2020*a* ▸) by cloning the cDNA encoding CA II in the pHL-sec vector (Aricescu *et al.*, 2006 ▸) and removing the secretion sequence. Expression of [^15^N]-His-labeled protein was carried out for 48 h in an expression medium reconstituted in the laboratory, in which [^13^C_6_,^15^N_3_]-histidine (Sigma–Aldrich) was added together with all of the other unlabeled components following the reported composition of high-glucose DMEM (Sigma) and was supplemented with 2% FBS and antibiotics. To ensure CA II metalation, zinc was supplemented immediately after transfection as ZnSO_4_ to a final concentration of 10 µ*M* in the expression medium. The concentration of CA II in the 150 µl lysate obtained from one 75 cm^2^ flask was estimated to be 150 ± 20 µ*M* by SDS–PAGE analysis by comparison with serial dilutions of a sample of purified CA II (Luchinat, Barbieri, Cremonini *et al.*, 2020*a* ▸,*b* ▸). Cells overexpressing CA II were treated with the compounds 48 h post-transfection by adding a concentrated stock solution of each compound (80 m*M* dissolved in DMSO) directly to 20 ml expression medium in the cell-culture flask to the desired final concentration. Experiments were performed by treating cells with varying amounts of each compound and by incubating them for different amounts of time, as specified in Section 3.

### 2.3. Closed-tube in-cell NMR sample preparation   

Samples for closed-tube in-cell NMR experiments were prepared as reported previously (Barbieri *et al.*, 2016 ▸). Briefly, transfected cells were detached with trypsin, suspended in DMEM + 10% FBS, washed once with PBS and resuspended in one pellet volume of NMR medium consisting of DMEM supplemented with 90 m*M* glucose, 70 m*M* HEPES and 20% D_2_O. The cell suspension was transferred into a 3 mm Shigemi NMR tube, which was gently spun to sediment the cells. In the final ∼150 µl pellet in the NMR tube the CA II concentration was ∼150 µ*M* (see Section 2.2). Cell viability before and after NMR experiments was assessed by a trypan blue exclusion assay. After the NMR experiments, the cells were collected and the supernatant was checked for protein leakage by NMR (Supplementary Fig. S1).

### 2.4. Production of agarose threads   

Cell samples in agarose threads were prepared as reported previously (Luchinat, Barbieri, Campbell *et al.*, 2020 ▸; Barbieri & Luchinat, 2021 ▸) by adapting an existing approach for encapsulating cells in NMR bioreactors (Burz *et al.*, 2019 ▸). Low-gelling agarose (Sigma–Aldrich) was dissolved at 1.5%(*w*/*v*) in PBS at 85°C, sterilized by filtration with a 0.22 µm filter, aliquoted in Eppendorf tubes and stored at 4°C. For sample preparation, one aliquot of solidified agarose was melted at 85°C and subsequently kept in solution at 37°C. A pellet of cells overexpressing CA II, collected from one 75 cm^2^ flask (∼3 × 10^7^ cells), was heated at 37°C for 15–20 s in a thermoblock. The cells were then resuspended in 450 µl agarose solution, carefully avoiding the formation of bubbles. The cell–agarose suspension was aspirated into chromatography PEEK tubing (outer diameter 1/16′′, inner diameter 0.75 mm) connected to a 1 ml syringe and was cooled to room temperature for 2 min. Threads were then cast into the flow-unit NMR tube, which contained an ∼5 mm-high bottom plug of 1.5% agarose gel (to place the cell sample within the active volume of the ^1^H NMR coil) and was prefilled with 100 µl PBS. The effective concentration of CA II in the flow-unit NMR tube was ∼40 µ*M* in 550 µl.

### 2.5. NMR bioreactor setup   

The NMR bioreactor employed in this study consists of a watertight flow unit based on the InsightMR flow-tube system (Bruker) compatible with standard 5 mm NMR probes; see Barbieri & Luchinat (2021 ▸) for a detailed description of the flow-unit and valve system. A programmable peristaltic pump (Reglo ICC Pump, Ismatec) with three independent channels was employed to provide a controlled flow of media at different ligand concentrations. Tygon 3350 tubing (outer diameter 0.9 mm, inner diameter 0.64 mm, three-stopper; PRO LIQUID GmbH) was used for all channels. The pump was connected to the flow unit through a four-way junction that allowed mixing of the output of up to three channels. Each channel was connected to a reservoir solution of un­labeled DMEM (Sigma–Aldrich, catalog No. D5648; powder, reconstituted in sterile-filtered Milli-Q H_2_O and supplemented with 2% FBS, 10 m*M* NaHCO_3_, antibiotics and 2% D_2_O, pH 7.4) containing one or two ligands at the concentrations specified in Table 1 ▸. During the bioreactor run, the timings and the flow rates of each channel were controlled from a PC connected to the pump using the pre-programmed operating mode (Table 1 ▸). The final flow (*i.e.* the sum of all channels) was kept constant at 0.1 ml min^−1^. The medium reservoir of each channel consisted of a 250 or 500 ml glass bottle kept at 37°C in a water bath. Each bottle was sealed with a steel headpiece with two hose nozzles, one connected to the corresponding pump channel through silicone tubing and the other connected to a 0.22 µm PTFE syringe filter for air intake.

### 2.6. In-cell NMR experiments   

Closed-tube in-cell NMR spectra were recorded at 310 K on a 900 MHz Bruker Avance NEO equipped with a 5 mm TCI CryoProbe. Bioreactor in-cell NMR spectra were recorded at 310 K using a Bruker Avance III 950 MHz equipped with a 5 mm TCI CryoProbe. 2D ^1^H–^15^N SOFAST-HMQC (Bruker pulse sequence sfhmqcf3gpph) spectra (Schanda & Brutscher, 2005 ▸) were recorded with frequency offsets of 4.7 p.p.m. (^1^H) and 172.5 p.p.m. (^15^N), spectral windows of 24 p.p.m. (^1^H) and 17 p.p.m. (^15^N), acquisition times of 33.6 ms (^1^H) and 19.5 ms (^15^N) and an interscan delay of 0.3 s, using the shaped pulses Pc9_4_120.1000 and Rsnob.1000 for selective ^1^H inversion and refocusing, respectively. The excitation width and offset were set to 5.5 and 13 p.p.m., respectively, for the selective excitation of the histidine H^δ1^/N^δ1^ and H^ɛ2^/N^ɛ2^ correlations of CA II. Shaped pulse lengths and power levels were automatically calculated (-DCALC_SP option in the pulse sequence). An apodization-weighted sampling scheme was introduced in the pulse program to further enhance the spectral sensitivity, in which the number of scans during the acquisition of the indirect dimension was scaled according to a square cosine bell function (Simon & Köstler, 2019 ▸). 256 initial scans (closed tube) or 64 initial scans (bioreactor) were employed in total durations for each spectrum of 51 min (closed tube) and 12 min 45 s (bioreactor). A single 2D spectrum was recorded for each closed-tube sample, while a series of 2D spectra was recorded during each bioreactor run for a total duration up to ∼60 h.

### 2.7. *In vitro* NMR experiments   

Samples of pure CA II (at the concentrations specified in Table 2 ▸) were placed in 5 mm NMR tubes and analyzed at 310 K on a 900 MHz Bruker Avance NEO equipped with a 5 mm TCI CryoProbe. 2D ^1^H–^15^N SOFAST-HMQC spectra with an apodization-weighted sampling scheme were recorded as described above, changing the following parameters: acquisition times of 47.1 ms (^1^H) and 25.8 ms (^15^N), 64 initial scans and a total duration of 16 min 43 s. 2D spectra were acquired in the absence of ligands and upon the addition of one or two ligands from stock solutions (80 m*M* dissolved in DMSO) at the final concentrations reported in Table 2 ▸.

### 2.8. NMR data analysis   

The 2D NMR spectra were processed in *TopSpin* 4.0 (Bruker) by applying zero filling on both dimensions and a square cosine bell apodization (SSB = 2) on the ^1^H dimension. For analysis of *in vitro* NMR spectra and closed-tube in-cell NMR spectra, well resolved signals arising from different CA II species were integrated using *TopSpin* 4.0. The relative fractions of CA II bound to each ligand were then obtained by dividing the integral for each species by the sum of the two. The bioreactor in-cell NMR spectra were analyzed as described previously (Luchinat, Barbieri, Campbell *et al.*, 2020 ▸; Barbieri & Luchinat, 2021 ▸) using the *MCR-ALS* 2.0 graphical user interface implemented in *MATLAB* (MathWorks; Juan & Tauler, 2006 ▸; Jaumot *et al.*, 2015 ▸). Briefly, 2D spectra were imported using the Read_Bruker_2D script provided by NMRFAM, University of Wisconsin-Madison (http://pine.nmrfam.wisc.edu/download_scripts.html). The spectral regions of interest were cut, converted to row vectors and stacked in a 2D array (time points × spectral intensities). In *MCR-ALS* 2.0, the number of components (*n* = 2) was evaluated by singular value decomposition, the initial estimation of pure spectra was made by purest variable detection, non-negativity constraints were applied both to rows (concentrations) and columns (spectra), and no further closure constraints or normalizations were applied. The fitting was run with a 0.01 convergence criterion and reached convergence after <30 iterations. To improve the *MCR-ALS* fitting, series of 2D spectra from different bioreactor runs, in which cells were treated with the same ligands, were joined and analyzed together. After the fitting, the relative fraction of CA II bound to each ligand was retrieved by averaging the values reached at the plateau after each step of the run (typically, 10–15 values were averaged for each step) and by dividing them by the sum of the averages for each species for each step.

### 2.9. Curve fitting   

Nonlinear curve fitting was performed in *OriginPro* 8 (OriginLab) to retrieve the *K*_d_ of the tested ligands from the *K*_d_ of the reference ligand. For competition binding experiments by *in vitro* NMR, the fraction of CA II bound to the tested ligand *F*_I_ = [EI]/[E_t_] as a function of the total concentration of reference ligand [L_t_] and tested ligand [I_t_] was fitted with the equation 
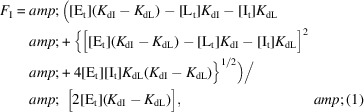
which is derived from the equilibrium equation of the two strong ligands L and I competing for binding to the protein E, 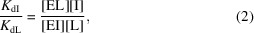
where *K*_dL_ and *K*_dI_ are the dissociation constants of L and I, respectively, assuming a pure competition mechanism:
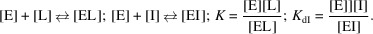



Equation (1) accounts for the depletion of free ligand upon binding and is correct as long as the concentration of free protein [E] is negligible, which is true for strong ligands when their sum is in molar excess with respect to the protein,
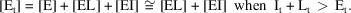



The correctness of this approximation is demonstrated by the lack of signals arising from free CA II in the 2D NMR spectra.

For in-cell NMR competition binding experiments, both in the closed tube and in the bioreactor, a simplified formula was used to fit the fraction of CA II bound to each ligand:
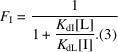



Equation (3) was obtained from equation (2) and can be used when the free ligand concentration is constant and known (*i.e.* in the NMR bioreactor) or when both ligands are in a large molar excess with respect to the protein (*i.e.* in the culture flask before harvesting the cells for closed-tube in-cell NMR), under the following approximation:
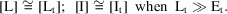



It can be shown that when 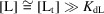 equation (3) becomes identical to the equation of the displacement value *F* reported previously for competition binding NMR experiments (equation 19 in Dalvit *et al.*, 2019 ▸).

## 3. Results   

### 3.1. Ligand binding to CA II *in vitro* and in cells   

CA II can be overexpressed in human cells at NMR-detectable levels and is free from interactions with slow-tumbling cellular components, and it therefore gives rise to well resolved signals in in-cell NMR spectra (Luchinat, Barbieri, Cremonini *et al.*, 2020*a* ▸). In addition, signals arising from the slow-exchanging hydrogens of the three zinc-binding histidines, His94 H^δ1^, His96 H^δ1^ and His119 H^ɛ2^, as well as His107 H^δ1^ located in the vicinity of the active site, fall in the region of the ^1^H NMR spectrum between 12 and 16 p.p.m. (Shimahara *et al.*, 2007 ▸; Vasa *et al.*, 2019 ▸), which is free from cellular background signals. It has previously been shown that the chemical shift changes in this spectral region induced by ligand binding allow protein–ligand interactions to be monitored both *in vitro* and in cells from 1D ^1^H NMR spectra (Luchinat, Barbieri, Cremonini *et al.*, 2020*a* ▸,*b* ▸; Luchinat, Barbieri, Campbell *et al.*, 2020 ▸). For protein-observed binding experiments, uniform [^15^N]-labeled CA II (*in vitro*) or [^15^N]-His-labeled CA II (in cells) was analyzed in order to reduce the signal overlap even further by separating the signals along the ^1^H and the ^15^N chemical shift dimensions.

**Figure 1:**
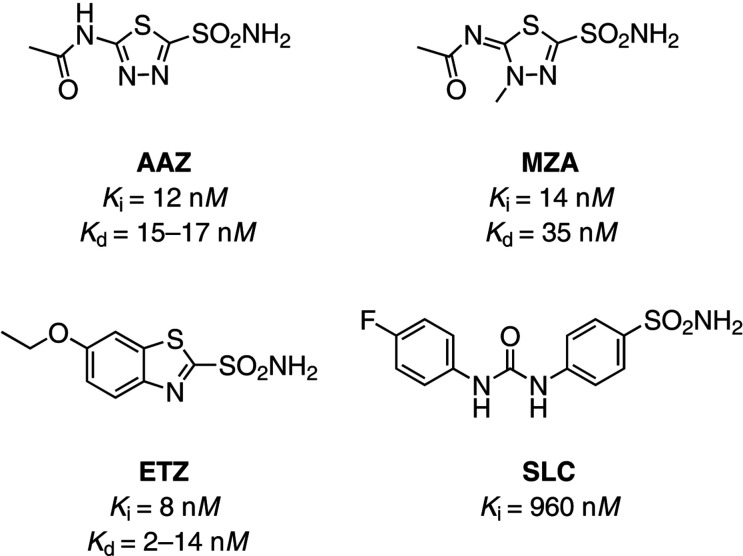
Chemical structures of the sulfonamide-derived compounds analyzed in this study. The *K*_i_ and *K*_d_ values previously reported *in vitro* for CA II are shown (see Table 3 ▸).

**Figure 2:**
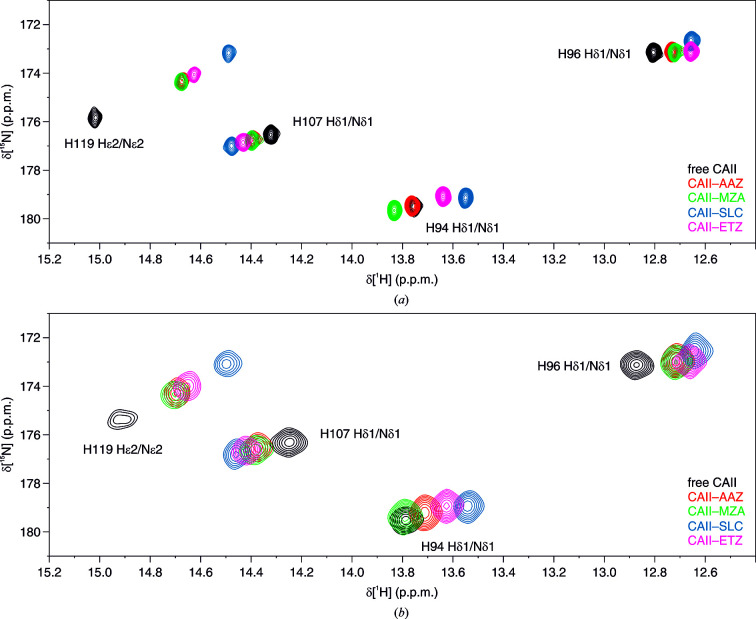
Overlay of ^1^H–^15^N NMR spectra of (*a*) pure [^15^N]-labeled CA II and (*b*) cells expressing [^15^N]-His-labeled CA II either in the absence of ligands (black) or bound to AAZ (red), MZA (green), SLC (blue) and ETZ (magenta). The signals of the free protein are labeled with the corresponding residue number and atom type (Shimahara *et al.*, 2007 ▸; Vasa *et al.*, 2019 ▸).

The binding of a set of sulfonamide derivatives to CA II was investigated through *in vitro* NMR (Fig. 1 ▸). Acetazolamide (AAZ) and methazolamide (MZA) are two approved drugs employed in the treatment of glaucoma, ethoxzolamide (ETZ) is a diuretic that inhibits CAs in proximal renal tubules, and SLC-0111 (SLC) is a CA inhibitor with high selectivity for the CA IX isoform and is currently in Phase Ib/II clinical trials as an anticancer/antimetastatic agent (McDonald *et al.*, 2020 ▸). These compounds have been extensively characterized and are known to inhibit CA II in the low-nanomolar to high-nanomolar range (Fig. 1; Supuran, 2008 ▸; Zubrienė *et al.*, 2009 ▸; Morkūnaitė *et al.*, 2015 ▸; Carta *et al.*, 2017 ▸; Linkuvienė, Talibov *et al.*, 2018 ▸). Fast 2D ^1^H–^15^N NMR spectra in the histidine spectral region recorded on recombinant CA II, either free or in the presence of a ligand, showed that the binding of each molecule caused different chemical shift perturbations, thus allowing the straightforward quantification of CA II bound to different ligands in competition binding experiments (Fig. 2 ▸ *a*). The same 2D ^1^H–^15^N NMR spectra recorded on human cells expressing [^15^N]-His-labeled CA II and treated with an excess of each ligand showed similar chemical shift changes, confirming that the compounds could penetrate the cells and bind quantitatively to intracellular CA II (Fig. 2 ▸ *b*).

### 3.2. Competition binding *in vitro*   

Competition binding experiments *in vitro* were carried out on samples of CA II containing MZA as a reference ligand at a constant concentration, in which the second ligand was added at increasing concentrations (Table 2 ▸). For the analysis of ETZ, which was found to have a much higher affinity than MZA (see below), it was chosen to keep ETZ at a constant concentration while varying the concentration of MZA. The fraction of CA II bound to each ligand in the mixture was quantified by signal integration and fitted with equation (1) to retrieve the *K*_d_ ratio of each ligand relative to that of MZA (Supplementary Fig. S2 and Table 3 ▸). The overall good quality of the fits confirmed that the histidine NMR signals are good reporters of the fraction of CA II bound to each ligand. The dissociation constant of AAZ, previously obtained by surface plasmon resonance (SPR; Linkuvienė, Talibov *et al.*, 2018 ▸) and nano-electrospray ionization (nano-ESI; Nguyen *et al.*, 2019 ▸), was taken as a reference value to calculate the absolute *K*_d_ of the other ligands (Table 3 ▸). Overall, the *K*_d_ values obtained with this method are in good agreement with those previously measured *in vitro*, whereas they deviate more from the *K*_i_ values of the same ligands measured by a CO_2_ hydration assay (Table 3 ▸). Such discrepancies have been reported previously for AAZ and some of the other ligands (Linkuvienė, Zubrienė *et al.*, 2018 ▸), and are likely to be intrinsic to the different type of assays employed (*i.e.* ligand binding versus enzyme inhibition) and of the different working conditions (*i.e.* enzyme concentration, buffer type and pH, CO_2_ partial pressure). However, ordering the ligands based on the *K*_d_ obtained by NMR gave the same result as ordering them by the *K*_i_ and the *K*_d_ determined previously, ETZ < AAZ < MZA < SLC, thus indicating that competition binding NMR can reliably assess relative ligand-binding affinities.

### 3.3. Competition binding by ’closed-tube’ in-cell NMR   

**Figure 3:**
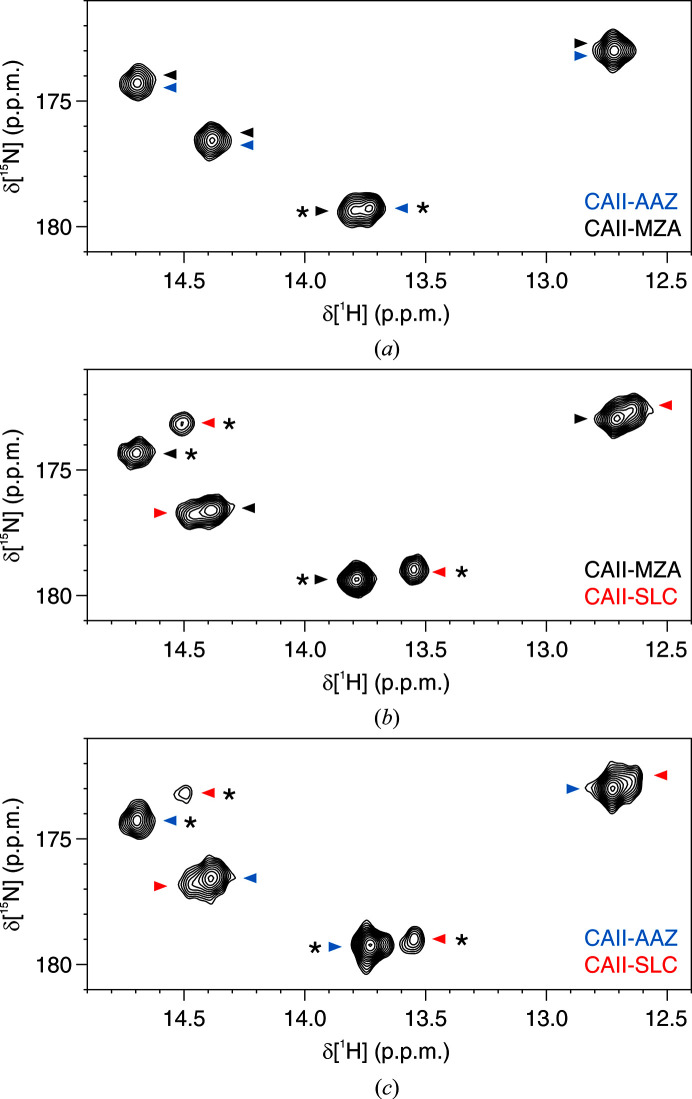
Closed-tube in-cell ^1^H–^15^N NMR spectra of cells treated with (*a*) 50 µ*M* AAZ + 100 µ*M* MZA, (*b*) 50 µ*M* MZA + 100 µ*M* SLC and (*c*) 50 µ*M* AAZ + 100 µ*M* SLC. Signals arising from CA II-bound AAZ, MZA and SLC are indicated with blue, black and red arrows, respectively. Only the least overlapped signals (marked with asterisks) were integrated for nonlinear fitting (see Supplementary Fig. S3).

Competition binding experiments were carried out in living cells by measuring the ligand-bound CA II fractions in different samples of cells expressing [^15^N]-His-labeled CA II. Each sample was treated with two ligands at a time, with each ligand at 50 or 100 µ*M* in the culture medium. The incubation time was 1 h for all samples, except for those containing 50 µ*M* AAZ, which were incubated for 2 h to compensate for the slow diffusion of AAZ through the plasma membrane (Luchinat, Barbieri, Cremonini *et al.*, 2020*a* ▸). After incubation, the cells were detached and ‘closed-tube’ in-cell NMR analysis by fast 2D ^1^H–^15^N NMR was carried out (Fig. 3 ▸). A total of 12 samples were analyzed, from which the fractions of CA II bound to each ligand were obtained by signal integration. ETZ was not investigated using this approach because at the concentrations employed it resulted in complete binding regardless of the second ligand concentration (data not shown). The data were globally fitted with equation (3) to retrieve the *K*_d_ of each ligand relative to the *K*_d(MZA)_ calculated by *in vitro* NMR (Supplementary Fig. S3 and Table 3 ▸). Overall, the binding affinities obtained by in-cell NMR data were similar to those determined *in vitro*, although, understandably, the goodness of fit was decreased in cells. This is partially caused by the lower resolution of in-cell NMR spectra with respect to those recorded *in vitro* due to the broader spectral lines caused by the higher viscosity of the cytosol and by additional inhomogeneous broadening induced by the cell sample (Luchinat *et al.*, 2021 ▸). This results in a higher signal overlap between different CA II adducts (especially between AAZ and MZA; see Fig. 3 ▸ *a*), leading to larger errors in the integration and subsequent analysis.

### 3.4. Competition binding by real-time bioreactor in-cell NMR   

**Figure 4:**
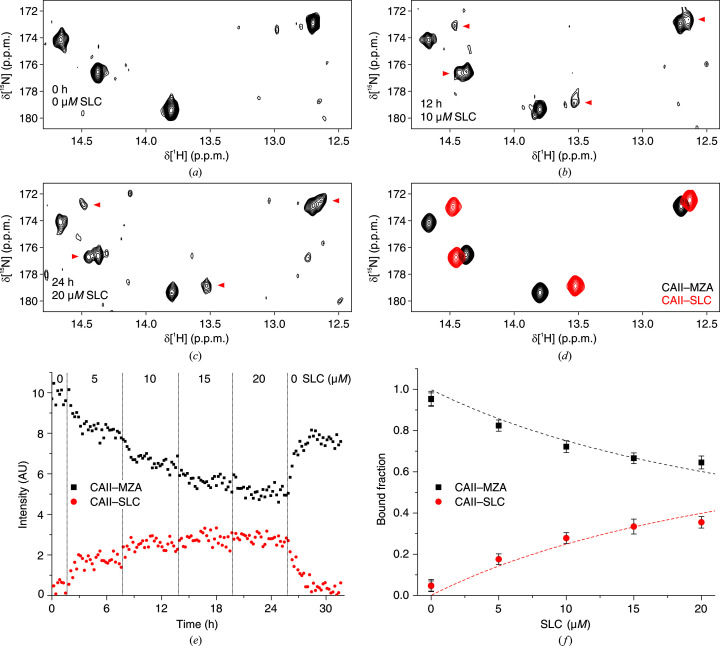
Bioreactor in-cell NMR of cells treated with constant 10 µ*M* MZA and increasing amounts of SLC (bioreactor run 1 in Table 1 ▸) and subsequent data analysis. (*a*–*c*) Representative ^1^H–^15^N NMR spectra at different time points and concentrations of SLC. Signals arising from SLC are marked with red arrows. (*d*) NMR spectra of the pure components, *i.e.* CA II–MZA (black) and CA II–SLC (red), reconstructed by *MCR-ALS*. (*e*) Concentration profiles of CA II–MZA (black squares) and CA II–SLC (red circles) obtained by *MCR-ALS*. (*f*) Bound fractions obtained from the plateau values after each step of the run plotted as a function of SLC concentration. Binding curves from nonlinear fitting are shown as dashed lines.

**Figure 5:**
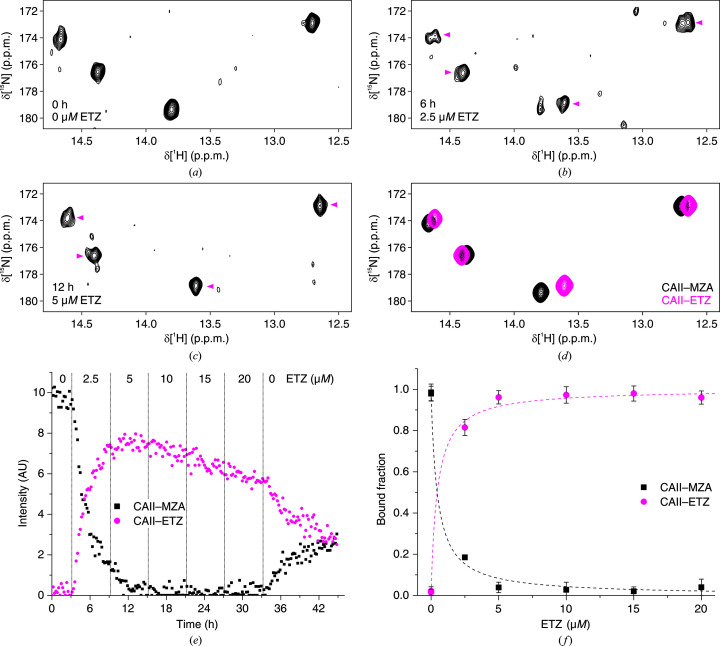
Bioreactor in-cell NMR of cells treated with constant 10 µ*M* MZA and increasing amounts of ETZ (bioreactor run 2 in Table 1 ▸) and subsequent data analysis. (*a*–*c*) Representative ^1^H–^15^N NMR spectra at different time points and concentrations of ETZ. Signals arising from ETZ are marked with magenta arrows. (*d*) NMR spectra of the pure components, *i.e.* CA II–MZA (black) and CA II–ETZ (magenta), reconstructed by *MCR-ALS*. (*e*) Concentration profiles of CA II–MZA (black squares) and CA II–ETZ (magenta circles) obtained by *MCR-ALS*. (*f*) Bound fractions obtained from the plateau values after each step of the run plotted as a function of ETZ concentration. Binding curves from nonlinear fitting are shown as dashed lines.

In ‘closed-tube’ in-cell NMR, each cell sample is treated with a mixture of ligands at given concentrations and is analyzed by NMR for a short time (typically <1 h) to avoid artifacts such as protein leakage resulting from cell death. This approach tends to become cost- and labor-intensive if many ‘points’ in ligand concentration are to be recorded for each tested ligand, requiring a large number of isotope-labeled human cell samples. Therefore, we evaluated an alternative approach for determining intracellular binding affinities, which makes use of the NMR bioreactor. In each bioreactor run, a single sample of cells expressing [^15^N]-His-labeled CA II was kept in the NMR spectrometer for up to 60 h under a steady flow of fresh medium, which preserved cell viability. By using a programmable multichannel peristaltic pump, the composition of the medium was changed over time in a stepwise manner in which the concentration of the tested ligand was incremented after each step while the concentration of the reference ligand was kept constant (Table 1 ▸). The duration of the steps was chosen to allow sufficient time for the ligands in the medium to penetrate the cells and to establish an equilibrium within the cells between free and bound to CA II. In the bioreactor experiments, AAZ was not analyzed as it was previously shown to diffuse through the plasma membrane approximately tenfold slower than MZA (Luchinat, Barbieri, Cremonini *et al.*, 2020*a* ▸; Luchinat, Barbieri, Campbell *et al.*, 2020 ▸). The displacement of MZA by SLC during each step was monitored by time-resolved 2D NMR (Figs. 4 ▸ *a*–4 ▸ *c*), followed by analysis by *MCR-ALS* to obtain the spectra of the pure components and the fractions of CA II bound to each ligand at each time point (Figs. 4 ▸ *d* and 4 ▸ *e*). The averaged plateau values at each step (Fig. 4 ▸ *f*) were fitted with equation (3) to retrieve the *K*_d_ of SLC relative to MZA. For ETZ, two bioreactor runs were performed with different doses of ETZ and durations for each step (Figs. 5 ▸ and 6 ▸). The fitting of the SLC data provided consistent *K*_d_ values with respect to those obtained by closed-tube in-cell NMR, whereas ETZ in the bioreactor appeared to bind CA II with a slightly lower affinity than in the closed-tube experiments (Table 3 ▸). The latter result may be due to the low external concentration of ETZ in the first steps of the competition experiment, which had to be used to compensate for the higher affinity for CA II with respect to MZA. As the rate of diffusion into the cells is proportional to the external ligand concentration (Luchinat, Barbieri, Campbell *et al.*, 2020 ▸), at very low concentrations ETZ may not have had sufficient time to establish equilibrium with MZA (see the initial steps in Figs. 5 ▸ *e* and 6 ▸ *e*). In general, this slow-diffusion effect could be mitigated by increasing the incubation time of the first steps or by increasing the concentration of both the reference and the tested ligand, thus improving the diffusion rate of both; however, in the latter case the effect of the prolonged treatment with a high concentration of ligands should be evaluated.

## 4. Discussion and conclusions   

Competition binding approaches are widely used *in vitro* to determine dissociation constants for protein–ligand inter­actions through many different techniques. These methods are especially useful when the intrinsic limitations of the technique employed do not allow the direct determination of the *K*_d_, typically in the case of high-affinity ligands. Here, we have shown that competition binding experiments in living human cells can be performed by protein-observed solution in-cell NMR spectroscopy, allowing the direct determination of intracellular *K*_d_ values in the nanomolar range relative to the *K*_d_ of a reference compound.

When the method was applied to CA inhibitors, the intracellular *K*_d_ values obtained by in-cell NMR were similar to the values obtained by NMR *in vitro*, which in turn are in good agreement with the *K*_d_ values determined *in vitro* using other techniques (Zubrienė *et al.*, 2009 ▸; Morkūnaitė *et al.*, 2015 ▸; Linkuvienė, Talibov *et al.*, 2018 ▸; Nguyen *et al.*, 2019 ▸) and, despite some discrepancies, quite consistent with the *K*_i_ values determined *in vitro* by activity assays (Supuran, 2008 ▸; Carta *et al.*, 2017 ▸). However, it should be stressed that the absolute value of all *K*_d_ values determined by competition binding is strictly dependent on the *K*_d_ of the reference compound. Therefore, in general, proper in-cell versus *in vitro* comparison of ligand-binding affinities requires the accurate determination of the intracellular absolute *K*_d_ of the reference compound, although this could prove to be a challenging task in the case of strong binding.

The two alternative approaches described here provided similar *K*_d_ values, and proved to be reliable for *K*_d_ values in a low-nanomolar to high-nanomolar range. Based on the sensitivity requirements for reliable quantitative analysis of in-cell NMR data, both closed-tube and bioreactor approaches should be applicable to freely tumbling protein targets of up to 30 kDa in size, as long as their effective concentration in the NMR tube is above ∼50 µ*M* (closed tube) or ∼15 µ*M* (bio­reactor). Concerning the affinity range of the screened ligands, reliable values should be obtained for *K*_d_ values falling within 1–2 orders of magnitude higher or lower than the *K*_d_ of the reference compound. Therefore, in principle, the range of affinities can be further extended by choosing a different reference ligand with higher or lower affinity for the target. The lower *K*_d_ limit is likely to depend on the dissociation rate of the reference compound. High-affinity ligands are slower to dissociate; thus, the time required to reach equilibrium with the competing ligand will increase. In the case of CAs, even the strongest inhibitors rarely take more than few hours to dissociate (Linkuvienė, Zubrienė *et al.*, 2018 ▸); therefore, this is not likely to impact on the applicability of the method. The upper *K*_d_ limit depends on the ligand toxicity: *K*_d_ values in the high-micromolar range will require higher ligand concentrations to quantitatively bind the target, and therefore high-micromolar-weight compounds with LC_50_ values in the millimolar range or lower are not likely to be compatible with this method. However, the *K*_d_ values of low-affinity ligands are better measured by direct binding, rather than by competition binding. Finally, the method requires that the ligands diffuse through the plasma membrane in a time range of minutes to hours. Therefore, as the rate of influx is linearly dependent on the external ligand concentration, slow-diffusing compounds showing toxicity at high concentrations will not be compatible, regardless of their affinity for the target.

From a practical standpoint, the bioreactor proved to be less labor-intensive and more cost-effective on the sample-preparation side with respect to the closed-tube approach, and data analysis by *MCR-ALS* proved to be more reliable in cases of severe spectral overlap of the NMR signals arising from the two ligand-bound protein species. In comparison, the closed-tube approach was overall easier to implement, more flexible (as it did not require planning all the ligand concentrations *a priori*, additional data points could be added at a later time) and suitable for slow-diffusing ligands (such as AAZ), as the incubation time occurs outside the NMR spectrometer. Therefore, the choice of approach depends on practical aspects such as the availability of the cells, the cost and effort required for each cell-sample preparation, the diffusion rate of the ligands and the spectral overlap between protein–ligand complexes.

In general, the method described here can be applied to intracellular soluble targets that can be observed by NMR and for which one or more signals in the NMR spectrum experience different chemical shift perturbations upon inter­action with different ligands. In principle, other labeling strategies can be employed, depending on which atoms/residues are to be observed. Importantly, the method is not limited to transiently transfected human cells and should be applicable to cells where isotope-labeled proteins are delivered through electroporation (Bekei, 2013 ▸; Theillet *et al.*, 2016 ▸) or other techniques (Inomata *et al.*, 2009 ▸; Ogino *et al.*, 2009 ▸), as well as to protein expressed in insect cells (Hamatsu *et al.*, 2013 ▸) and bacterial cells (DeMott *et al.*, 2018 ▸; Siegal & Selenko, 2019 ▸). In addition to protein targets, ligand affinity towards other types of targets, such as DNA and RNA, can also be investigated (Krafcikova *et al.*, 2019 ▸; Broft *et al.*, 2021 ▸). Furthermore, with a more complete characterization of the intracellular binding kinetics of the reference compound, competition binding/unbinding curves obtained by real-time bioreactor in-cell NMR could be fitted according to the drug-target residence-time model (Copeland, 2016 ▸), which considers the lifetime of the drug-target complex as a more reliable parameter for assessing drug potency in cells and tissues. In theory, the in-cell NMR competition binding approach should allow determination of the in-cell off-target binding activity of the compounds investigated, as the other cellular proteins that compete for binding will subtract ligand from the target, leading to a higher apparent dissociation constant. However, in practice treating the cells with a large excess of ligand at micromolar concentrations will saturate most of the off-target binding sites, thereby masking the competition from off-target binding sites. Lower intracellular levels of target will partly overcome this limitation, at the expense of a decrease in the sensitivity of the method. Approaches relying on ligand-observed in-cell NMR (Primikyri *et al.*, 2018 ▸; Bouvier *et al.*, 2019 ▸), which have yet to be fully developed, could prove to be more useful to study such phenomena. Eventually, we expect that intracellular *K*_d_ determination by competition binding through in-cell NMR will provide important insights into the efficacy of candidate drugs towards their designated intra­cellular target, which is crucial in the identification of more promising compounds before moving to preclinical and clinical phases, and for the development of more effective drugs with fewer side effects.

## Supplementary Material

Supplementary Figures. DOI: 10.1107/S2059798321009037/qt5006sup1.pdf

## Figures and Tables

**Figure 6:**
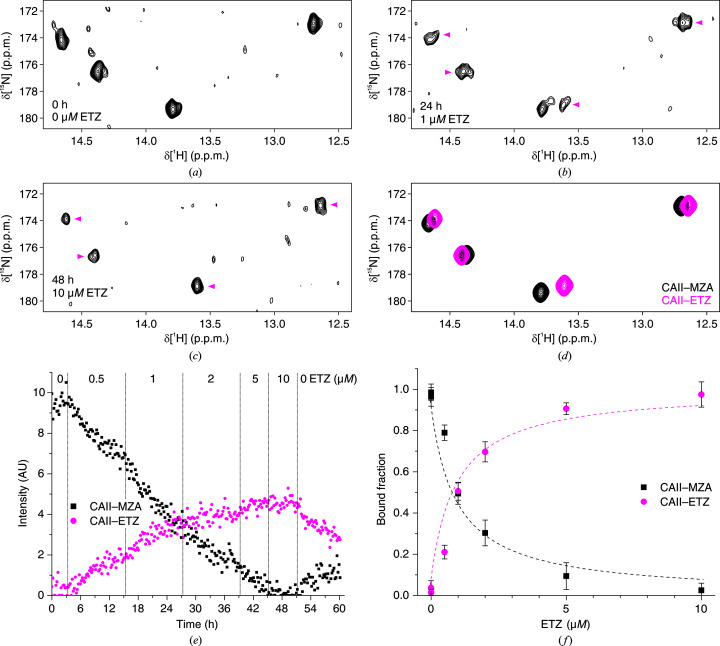
Bioreactor in-cell NMR of cells treated with constant 10 µ*M* MZA and increasing amounts of ETZ (bioreactor run 3 in Table 1 ▸) and subsequent data analysis. (*a*–*c*) Representative ^1^H–^15^N NMR spectra at different time points and concentrations of ETZ. Signals arising from ETZ are marked with magenta arrows. (*d*) NMR spectra of the pure components, *i.e.* CA II–MZA (black) and CA II–ETZ (magenta), reconstructed by *MCR-ALS*. (*e*) Concentration profiles of CA II–MZA (black squares) and CA II–ETZ (magenta circles) obtained by *MCR–ALS*. (*f*) Bound fractions obtained from the plateau values after each step of the run plotted as a function of ETZ concentration. Binding curves from nonlinear fitting are shown as dashed lines.

**Table:** Bioreactor run 1. Channel 1, 10 µ*M* MZA; channel 2, 10 µ*M* MZA + 20 µ*M* SLC.

Step	Length (h)	MZA (µ*M*)	SLC (µ*M*)	Channel 1 flow rate (µl min^−1^)	Channel 2 flow rate (µl min^−1^)
1	6	10	0	100	0
2	6	10	5	75	25
3	6	10	10	50	50
4	6	10	15	25	75
5	6	10	20	0	100
6	6	10	0	100	0

**Table:** Bioreactor run 2. Channel 1, 10 µ*M* MZA; channel 2, 10 µ*M* MZA + 20 µ*M* ETZ.

Step	Length (h)	MZA (µ*M*)	ETZ (µ*M*)	Channel 1 flow rate (µl min^−1^)	Channel 2 flow rate (µl min^−1^)
1	6	10	0	100	0
2	6	10	2.5	87.5	12.5
3	6	10	5	75	25
4	6	10	10	50	50
5	6	10	15	25	75
6	6	10	20	0	100
7	12	10	0	100	0

**Table:** Bioreactor run 3. Channel 1, 10 µ*M* MZA; channel 2, 10 µ*M* MZA + 10 µ*M* ETZ.

Step	Length (h)	MZA (µ*M*)	ETZ (µ*M*)	Channel 1 flow rate (µl min^−1^)	Channel 2 flow rate (µl min^−1^)
1	6	10	0	100	0
2	12	10	0.5	95	5
3	12	10	1	90	10
4	12	10	2	80	20
5	6	10	5	50	50
6	6	10	10	0	100
7	12	10	0	100	0

**Table 2:** Ligand concentrations after each addition in the competition binding experiments *in vitro* The CA II concentration for each experiment is reported.

Experiment 1: 120 µ*M* CA II			Experiment 2: 30 µ*M*CA II			Experiment 3: 30 µ*M* CA II		
Addition	MZA (µ*M*)	AAZ (µ*M*)	Addition	MZA (µ*M*)	AAZ (µ*M*)	Addition	MZA (µ*M*)	ETZ (µ*M*)
1	300	0	1	50	0	1	0	50
2	300	100	2	50	25	2	100	50
3	300	200	3	50	50	3	200	50
4	300	300	4	50	75	4	300	50
5	300	400	5	50	100	5	400	50
			6	50	150			
			7	50	200			

**Table 3:** Inhibitory constants (*K*_i_) and dissociation constants (*K*_d_) reported in the literature, *K*_d_ obtained *in vitro* by NMR and *K*_d_ obtained by in-cell NMR in a closed tube and in a bioreactor Reference *K*_d_ values for each column are shown in bold. For literature *K*_i_ and *K*_d_ values, the technique used is indicated in the footnotes.

	*In vitro* (literature)		*In vitro* NMR	In-cell NMR	
Ligand	*K*_i_ (n*M*)	*K*_d_ (n*M*)	*K*_d_ (n*M*)	*K*_d_, closed-tube (n*M*)	*K*_d_, bioreactor (n*M*)
MZA	14†	35‡	37 ± 1	**37** §	**37** §
AAZ	12†	17‡/15¶/15††	**15** ¶ ††	18 ± 1	n.d.
SLC	960‡‡	n/a	79 ± 3	95 ± 8	111 ± 11
ETZ	8†	14‡/2§§	0.45 ± 0.02	n.d.	1.6 ± 0.3¶¶/3.7 ± 0.6†††

† From CO_2_ hydration assay (Supuran, 2008 ▸).

‡ From isothermal titration calorimetry (Morkūnaitė *et al.*, 2015 ▸).

§ From competition with AAZ *in vitro*.

¶ From SPR (Linkuvienė, Talibov *et al.*, 2018 ▸).

†† From nano-ESI (Nguyen *et al.*, 2019 ▸).

‡‡ From CO_2_ hydration assay (Carta *et al.*, 2017 ▸).

§§ From thermal shift assay (Zubrienė *et al.*, 2009 ▸).

¶¶ From bioreactor run 2.

††† From bioreactor run 3.
