# Supplementary material for: Determination of intracellular protein–ligand binding affinity by competition binding in-cell NMR
Source: Acta Crystallogr D Struct Biol. 2021 Sep 27;77(Pt 10):1270–81. doi: 10.1107/S2059798321009037 (PMC8489230; doi:10.1107/S2059798321009037)
Supplement: Supplementary file 1 [file d-77-01270-sup1.pdf]

STRUCTURAL BIOLOGY

Volume 77 (2021)

Supporting information for article:

Determination of intracellular protein–ligand binding affinity by competition binding in-cell NMR

Enrico Luchinat, Letizia Barbieri, Matteo Cremonini, Matteo Pennestri, Alessio Nocentini, Claudiu T. Supuran and Lucia Banci

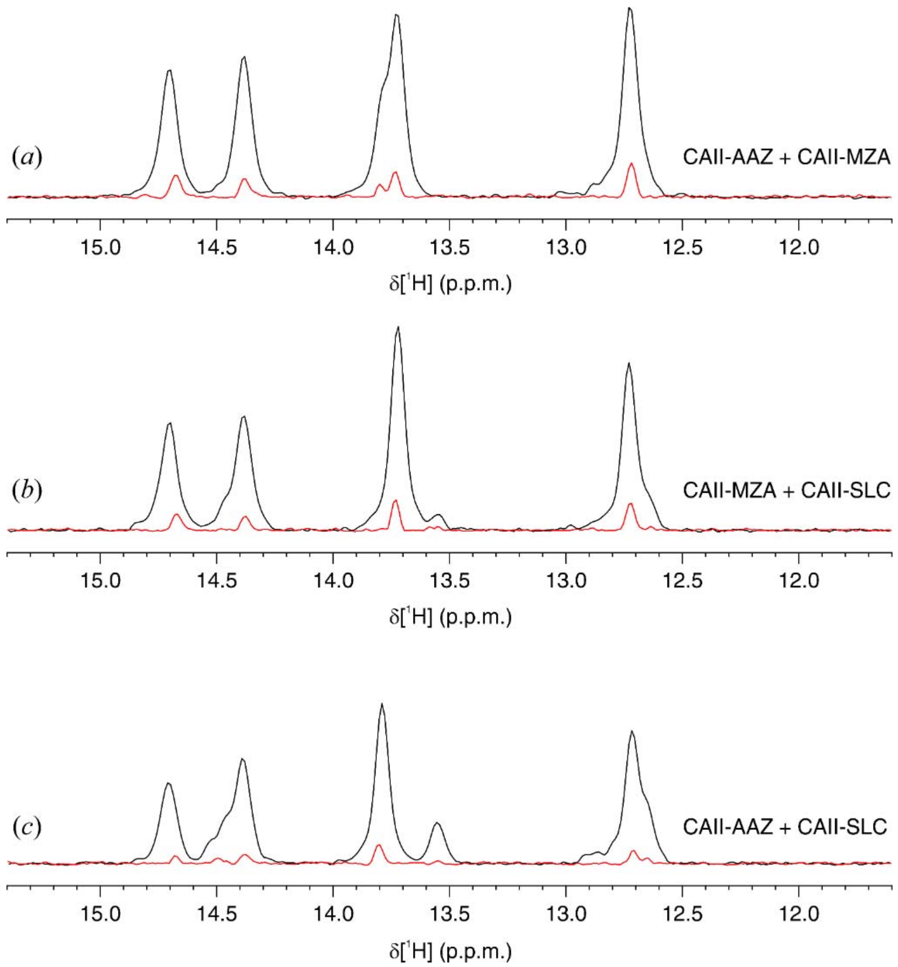

Figure S1 ^{1}H projections of ^{1}H-^{15}N NMR spectra recorded on intact cells in closed tube (black) treated with (*a*) 50 µM AAZ + 100 µM MZA; (*b*) 50 µM MZA + 100 µM SLC; (*c*) 50 µM AAZ + 100 µM SLC, and on the corresponding supernatants (red) after ~1 hour of in-cell NMR experiments. Protein leakage, estimated by total peak area, amounts to 8% (*a*), 5% (*b*), and 7% (*c*).

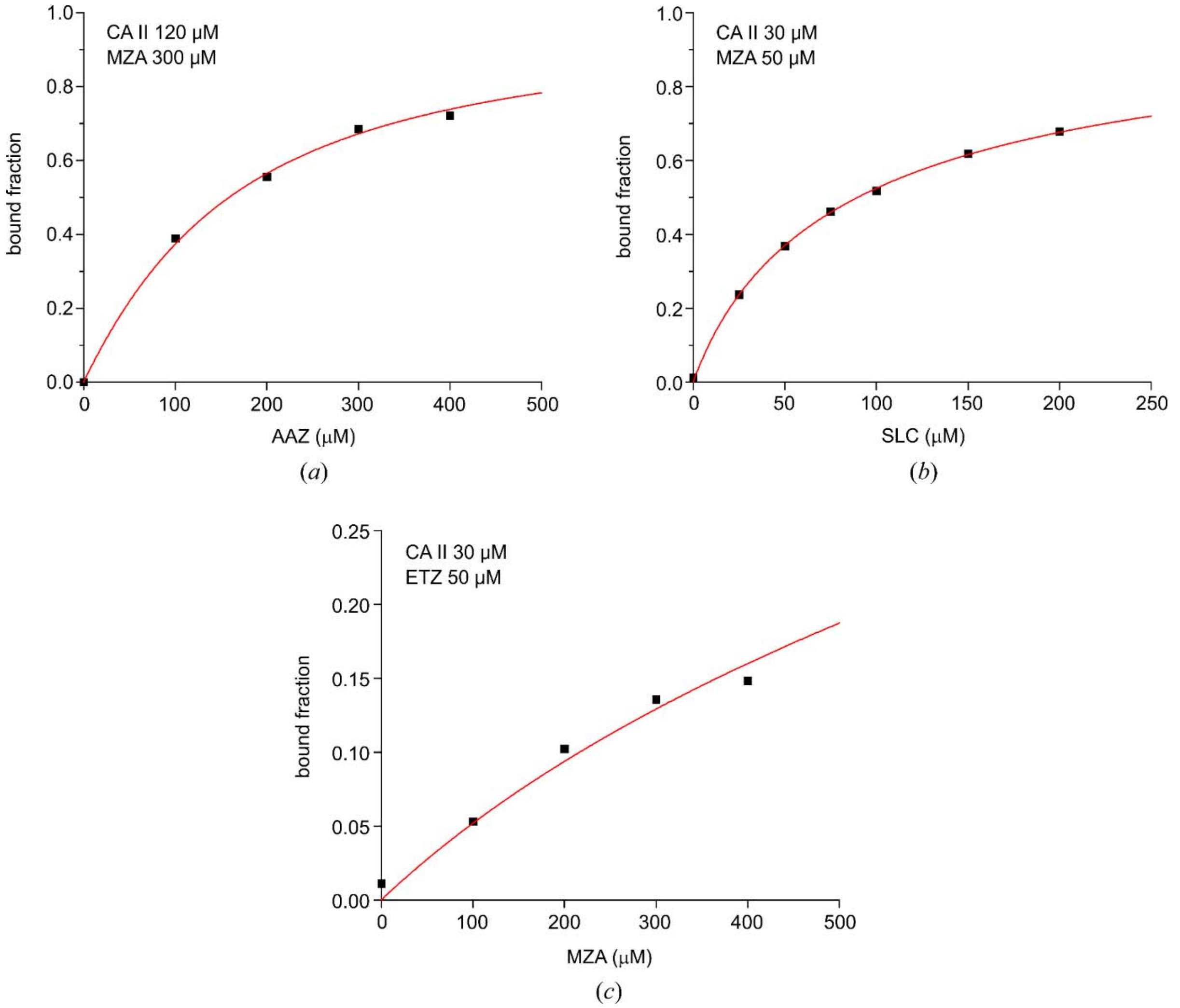

Figure S2 Non-linear curve fitting of competition binding experiments *in vitro*. (*a*) fraction of CA II bound to AAZ in the presence of MZA; (*b*) fraction of CA II bound to SLC in the presence of MZA; (*c*) fraction of CA II bound to MZA in the presence of ETZ. K_d values obtained from the fittings are reported in Table 3.

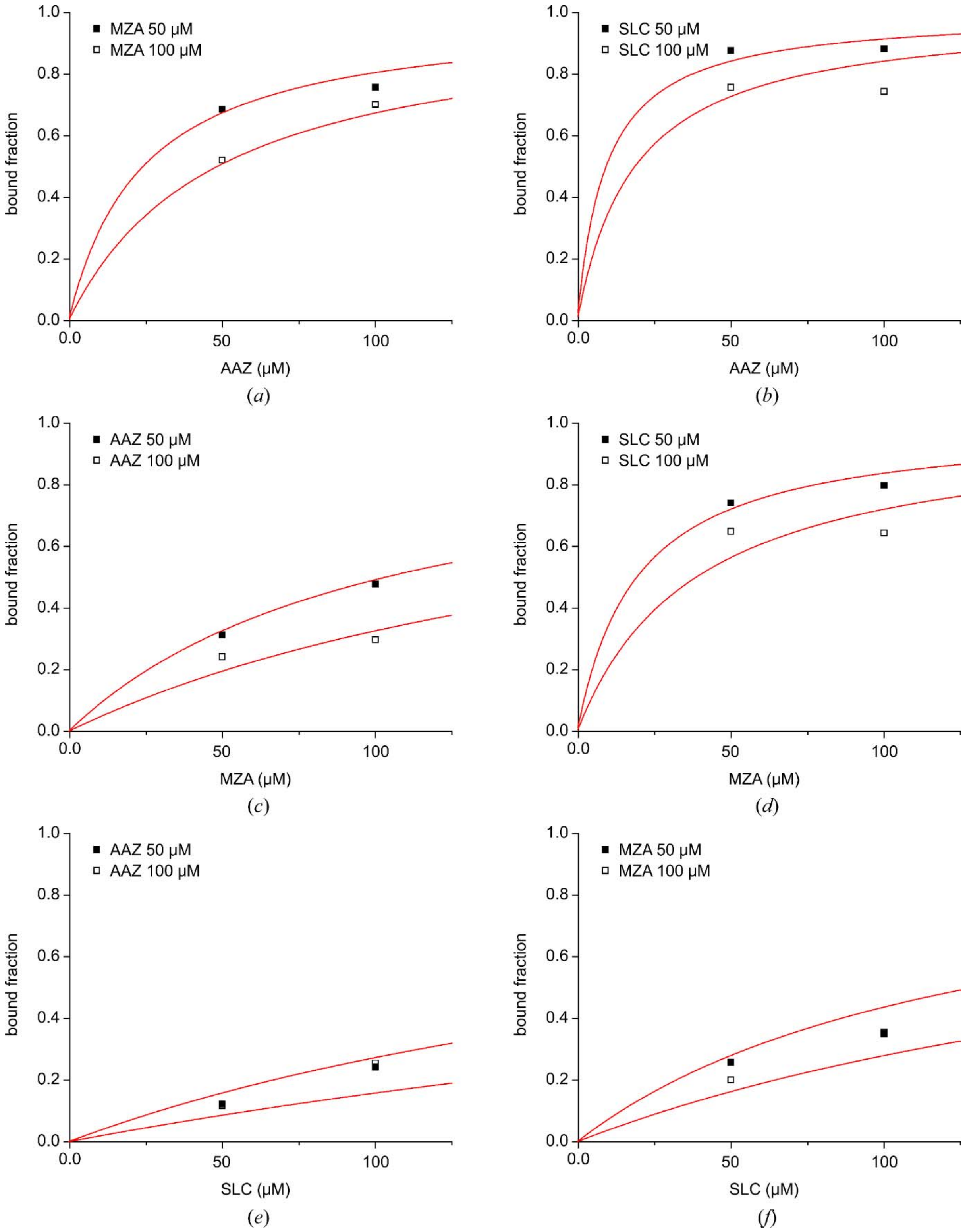

Figure S3 Global non-linear curve fitting of competition binding experiments by closed-tube in-cell NMR (see Figure 3). (*a*,*b*) fraction of CA II bound to AAZ in the presence of MZA (*a*) or SLC (*b*); (*c*,*d*) fraction of CA II bound to MZA in the presence of AAZ (*c*) or SLC (*d*); (*e*,*f*) fraction of CA II bound to SLC in the presence of AAZ (*e*) or MZA (*f*). K_d values obtained from the fittings are reported in Table 3.
